# Characterisation of manganese peroxidase and laccase producing bacteria capable for degradation of sucrose glutamic acid-Maillard reaction products at different nutritional and environmental conditions

**DOI:** 10.1007/s11274-018-2416-9

**Published:** 2018-02-02

**Authors:** Vineet Kumar, Ram Chandra

**Affiliations:** Department of Environmental Microbiology, School for Environmental Sciences, Babasaheb Bhimrao Ambedkar Central University, Vidya Vihar, Raebareli Road, Lucknow, Uttar Pradesh 226025 India

**Keywords:** Melanoidins, Manganese peroxidase, Laccase, Metabolites, GC–MS analysis

## Abstract

Maillard reactions products (MRPs) are a major colorant of distillery effluent. It is major source of environmental pollution due to its complex structure and recalcitrant nature. This study has revealed that sucrose glutamic acid-Maillard reaction products (SGA-MRPs) showed many absorption peaks between 200 and 450 nm. The absorption maximum peak was noted at 250 nm in spectrophotometric detection. This indicated the formation of variable molecular weight Maillard products during the SGA-MRPs formation at high temperature. The identified aerobic bacterial consortium consisting *Klebsiella pneumoniae* (KU726953), *Salmonella enterica* (KU726954), *Enterobacter aerogenes* (KU726955), *Enterobacter cloaceae* (KU726957) showed optimum production of MnP and laccase at 120 and 144 h of growth, respectively. The potential bacterial consortium showed decolourisation of Maillard product up to 70% in presence of glucose (1%), peptone (0.1%) at optimum pH (8.1), temperature (37 °C) and shaking speed (180 rpm) within 192 h of incubation. The reduction of colour of Maillard product correlated with shifting of absorption peaks in UV–Vis spectrophotometry analysis. Further, the changing of functional group in FT-IR data showed appearance of new peaks and GC–MS analysis of degraded sample revealed the depolymerisation of complex MRPs. The toxicity evaluation using seed of *Phaseolus mungo* L. showed reduction of toxicity of MRPs after bacterial treatment. Hence, this study concluded that developed bacterial consortium have capability for decolourisation of MRPs due to high content of MnP and laccase.

**Electronic supplementary material:**

The online version of this article (10.1007/s11274-018-2416-9) contains supplementary material, which is available to authorized users.

## Introduction

Melanoidins are complex, dark coloured, amino-carbonyl polymeric compounds with high molecular weight, formed by non-enzymatic browning Maillard reaction (MR) during several industrial processes (Hayase 2000; Echavarría et al. 2014; Bharagava and Chandra 2010). The major sources of melanoidins in environment are sugarcane-molasses based distilleries and fermentation industries all over the world. Due to its complex unknown organic pollutants and high total dissolved solids (TDS), the safe disposal of distillery waste is a paramount challenge for industrialist (Chandra and Kumar 2017). The toxicity of distillery waste is well documented in terrestrial and aquatic environment due to presence of heavy metals and recalcitrant organic compounds (Chandra et al. 2008a, b; Hemavanthi et al. 2015). The majority of molasses-based distilleries are located in tropical and subtropical regions of world and generate ~ 12–15 liters of effluent per liter of alcohol production. Currently, there are more than 397 distilleries are operating in India producing approx. 3.25 × 10^10^ liters of ethanol and generating 40.90 × 10^15^ liters of effluent annually (AIDA 2016).

Various physico-chemical methods such as column filtration (Satyawali and Balakrishnan 2008), flocculation (Liang et al. 2009a), adsorption (Onyango et al. 2011), chemical precipitation or coagulation (Chandra and Singh 1999; Liang et al. 2009b), UV/H_2_O_2_ treatment (Dwyer and Lant 2008) and ozone oxidation (Kim et al. 1985) has been reported for removal and decolourisation of distillery effluent, but these techniques are not feasible at large scale due to high cost and blockage of filtration devices. Moreover, due to high TDS it generates huge amount of toxic sludge and other secondary pollutants (Liang et al. 2009b; Chandra et al. 2008a). In addition, the conventional anaerobic digestion and activated sludge processes are effective in removing the biochemical oxygen demand (BOD) and chemical oxygen demand (COD) from effluent upto certain extent only. But, the color of distillery effluent converts darker with higher TDS after anaerobic treatment due to complexation of melanoidins. Therefore, extended aeration of distillery effluent does not change its physico-chemical properties even after aeration.

Hence, in recent years, the biological approaches with microbial decolourisation process of effluent for optimisation of various parameters at laboratory conditions have drawn attention of various workers world over to explore the feasible technology because it may lead eco-sustainable and cost effective alternative to chemical methods (Kaushik et al. 2010). Under biological treatment process using fungi such as *Geotrichum candidum* (Kim and Shoda 1999) *Flavadon flavus* (Raghukumar and Rivonkar 2001), *Phanerochaete chrysosporium* (Thakkar et al. 2006), *Trametes* sp. (Gonzalez et al. 2000), *Coriolus hirsutus* (Miyata et al. 2000), *Pleurotus florida, Aspergillus flavus* (Pant and Adholeya 2009a), *Neurospora intermedia* (Kaushik and Thakur 2013), *Fusarium verticillioides* (Pant and Adholeya 2009b) and yeast *Citeromyces* sp., *Candida tropicalis* (Tiwari et al. 2012), and *Candida glabrate* (Mahgoub et al. 2016) have been reported for melanoidins degradation using different category of melanoidins. The degradation and decolourisation of melanoidins by fungus has been reported more effective due to prevalence of ligninolytic enzymes, which metabolizes melanoidins as a sole carbon and nitrogen sources (Miyata et al. 2000; González et al. 2008). But, the large scale applications of these techniques have own constraint due to slow growth cycle, huge spore formation, low pH range (3.0–5.0), and adverse submerged aquatic environment for growth of fungus (Arimi et al. 2014). Therefore, bacteria are promising alternative for higher decolourisation due to its faster growth rate, higher environmental adaptability and high metabolizing capability of melanoidins by ligninolytic enzyme activity (Bharagava et al. 2009; Yadav et al. 2011). Some worker have reported the bacterial decolourisation and degradation of sugarcane molasses melanoidins as well as model melanoidins both (Kumar and Chandra 2006; Chandra et al. 2009; Bharagava and Chandra 2009). But, due to complex nature of melanoidins in sugarcane molasses with mixture of various other organic compounds it shows variable absorption range which makes more difficult to understand the mechanism of melanoidins decolourisation and characterization of its metabolic products. But, most of degradation/decolourisation process of molasses-melanoidins is reported at 475 nm only based on purified melanoidins absorption maxima through dialysis process with specific molecular weight, while the molasses-melanoidins contains mixture of Maillard products (i.e. initial, intermediate and advanced stages with variable molecular weight). Therefore, prior to attempting the degradation and decolourisation of distillery effluent, the degradation of model melanoidins with mixture of complex Maillard reaction products (MRPs) should be evaluated for its degradability. But, no any such study has been reported so far for assessment of melanoidins degradation as whole to assess the capability of bacterial consortium. Moreover, recent studies have revealed the preferential role of MnP for melanoidins depolymerisation followed by laccase induction in bacterial degradation (Yadav and Chandra 2012, 2013). The role of bacterial manganese peroxidase (MnP) and laccase for melanoidins degradation is less reported.

Therefore, the main objective of this study was to investigate the MnP and laccase producing bacterial consortium capability for the degradation and decolourisation of complex sucrose glutamic acid-Maillard reaction products (SGA-MRPs), which are predominantly present in sugarcane juice composition which generate the complex of molasses melanoidins due to thermal degradation reaction at subsequent stage (Walford 1996; Eggleston and Vercellotti 2000). Further, the change in absorption peaks (200–700 nm) by UV–Vis spectrophotometric analysis has been correlated for their structural changes and reduction in color by investigation through fourier transform-infrared spectroscopy (FT-IR), high performance liquid chromatography (HPLC), and gas chromatography–mass spectrometry (GC–MS) analysis. Furthermore, to evaluate the stability of potential bacterial consortium at variable nutritional and environmental conditions was assessed for optimum degradation and decolourisation of SGA-MRPs. The reduction of toxicity after bacterial degradation was also evaluated by using seed germination test of *Phaseolus mungo* L.

## Materials and methods

### Distillery sludge sample collection, isolation and screening of melanoidins tolerant, MnP, and laccase producing bacterial strains

The sludge sample was collected from the dumping site of M/s Unnao Distilleries and Breweries, located in Unnao, Uttar Pradesh (26°320″N, 80°30′0″E), India for isolation of potential bacterial strains. For isolation of bacterial strains, 10 g of sludge sample was transferred to a 250 mL Erlenmeyer flask containing 100 mL sterile GPM broth (g L^−1^; glucose 1%, peptone 0.1%, MgSO_4_·7H_2_O 0.05%, K_2_HPO_4_ 0.1%). The flasks were incubated at 37 ± 1 °C in a refrigerated incubator shaker (Orbitek, Scigenic Biotech, India) at 120 rpm for 7 days. The flask containing the sample showing the decolourisation was selected. Subsequently, an aliquot (1.0 mL) was taken serially diluted in order of 10^−2^, 10^−3^, 10^−4^, 10^−5^, and 10^−6^, then after diluted sample (0.1 mL) was spread on GPM agar medium plates. Further, the bacterial colonies showing clear zone on agar plates were purified on GPM agar plate by streak plate method.

To investigate, the bacterial tolerance on melanoidins purified bacterial strains were spread on pre-sterilised GPM agar medium plate amended with different concentration of molasses melanoidins (1000, 1500, 2000, 2500, 3000, and 3500 mg L^−1^ w/v). Each plate was incubated at 37 ± 1 °C for 48 h and observed for growth of tolerant bacterial colonies. The bacterial strains tolerant to high concentration of melanoidins were selected and screened for MnP and laccase activity by plate assay method. For enzyme screening, the substrate used for MnP (EC 1.11.1.13) was phenol red (Lobachemie, India) (Pangallo et al. 2007), while laccase (EC 1.10.3.2) activity was detected in presence of guaiacol (Sigma-Aldrich, St. Louis, MO, USA) as a substrate in B&K agar medium containing (g L^−1^) dextrose 1%, peptone 0.5%, beef extract 0.3%, NaCl 0.5%, and CuSO_4_ (1 mM) (D’Souza et al. 2006). The conversion of dark pink to yellow colour indicated the presence of MnP activity and reddish brown colour zones in the medium indicated the presence of laccase activity.

### Identification of bacterial strains

#### Morphological and biochemical characterisation

The isolated bacterial strains were identified based on morphological and biochemical characteristics by using the standard procedure described by Barrow and Feltham (2003).

#### Molecular characterisation

The isolated potential MnP and laccase producing bacterial strains were characterized by 16S rRNA gene sequence analysis. The total genomic DNA from the overnight growth culture of bacterial consortium was extracted using the method described by Kapley et al. (2001). The PCR amplicon were electrophoresed through 1.2% (w/v) agarose gel in 1× TAE buffer using 1 Kb DNA ladder (Merck Biosciences, India), then visualized by staining with ethidium bromide. Finally, the amplified 16S rDNA gene amplicon were purified from gel using a PCR cleanup kit (Merck Biosciences, India) and sequenced using 1492R and 27F eubacterial primer in an automated DNA sequencer. A phylogenetic tree was generated using the MEGA software (version 6.0) by the Neighbor-Joining method (Tamura et al. 2013).

### Maillard products decolourisation and degradation studies

#### Preparation and physico-chemical analysis of Maillard products

SGA-MRPs were prepared following the method described by Bharagava et al. (2009). Briefly, 1.0 M sucrose, 1.0 M glutamic acid, and 0.5 M sodium carbonate were dissolved in 1.0 L of doubled distilled water and the pH of the reaction mixture was adjusted to 8.0 with 1 N NaOH. After this, the solution was refluxed at 110 °C for 8 h. The prepared SGA-MRPs were diluted to 10% of its original concentration using distilled water to obtain the OD 3.5 ± 0.02 at 250 nm. This prepared SGA-MRPs solution was used for physico-chemical and decolourisation studies. The physico-chemical parameters (i.e. color, pH, BOD, COD, TDS, etc.) of SGA-MRPs were analysed as per standard methods (APHA 2012).

#### Construction of bacterial consortium for decolourisation studies

A loopful pure culture of isolated bacteria strains IITRCS01, IITRCS06, IITRCS07, and IITRCS11 was transferred to a 50 mL modified GPM broth medium supplemented with K_2_HPO_4_ (0.1%), MgSO_4_·7H_2_O (0.05%) and molasses melanoidins (1000 mg L^−1^) and incubated for 24 h in continuous shaking condition (125 rpm) at 37 ± 1 °C. After 24 h, the equal volume of each bacterial strains containing cell density 3.1 × 10^4^ cells mL^−1^ was used for preparation of different bacterial consortium. The bacterial strains IITRCS01, IITRCS06, IITRCS07, and IITRCS11 were taken in the various combinations i.e. in the ratio of 1:1:1:1, 1:2:1:1, 1:2:2:1, 1:2:2:2, 2:1:1:2; 2:2:1:1, 2:2:2:1 and 2:2:2:2 for optimal decolourisation of SGA-MRPs.

#### Decolourisation assay

The decolourisation experiment was carried out in Erlenmeyer flasks (250 mL) containing 100 mL of previously prepared (as mentioned in “Maillard products decolourisation and degradation studies” section) sterile SGA-MRPs solution supplemented with (glucose 1%, peptone 0.1%, K_2_HPO_4_ 0.1%) and MgSO_4_·7H_2_O (0.05% g L^−1^) and 1.0 mL of trace elements solution containing CuSO_4_·5H_2_O (0.003%), MnCl_2_·4H_2_O (0.001%), ZnSO_4_·7H_2_O (0.001%), and CaCl_2_·2H_2_O (0.002%), and pH was adjusted to 7.0 with 1 N NaOH (Bharagava et al. 2009). The flasks were inoculated with 1% (v/v) over night grown culture of bacterial consortium (OD 0.24) and incubated at 37 ± 1 °C under shaking flask condition (125 rpm) for 192 h. The decolourisation of SGA-MRPs was monitored by measuring the change in absorbance maxima of the melanoidins at 250 nm after scanning of absorbance maxima by using a UV–Vis spectrophotometer (Evoluation-201, Thermo Scientific, USA). Simultaneously, the un-inoculated flask contain same medium and condition was used as control. The % decolorization was calculated as

$${\text{Decolourisation }}(\% )=\frac{{{\text{initial absorbance (}}{{\text{A}}_0}) - {\text{final absorbance (}}{{\text{A}}_1}{\text{)}}}}{{{\text{initial absorbance (}}{{\text{A}}_0}{\text{)}}}} \times 100$$

### Optimisation of culture conditions for decolourisation and degradation of SGA-MRPs

#### Optimisation of nutritional parameters

The effect of different carbon source viz. sucrose, fructose, glucose, xylose, lactose, and starch at 1.0% (w/v) were evaluated for the SGA-MRPs decolourisation and degradation. In another experiment, different organic and inorganic nitrogen sources viz. peptone, beef extract, yeast extract, sodium sulphate, ammonium chloride and urea were added into the GPM medium at 0.5% (w/v) concentration. The effect of carbon and nitrogen sources were optimised for decolourisation and degradation SGA-MRPs at different concentration viz. 0.1–1.0 (w/v).

#### Optimisation of environmental conditions

To evaluate the effect of different environmental conditions the same experiment was carried out at different temperature (25–50 °C), pH (4–12), and shaking speed (100–220 rpm).

### Evaluation of bacterial growth, biomass, and scanning electron microscopic (SEM) observation of bacterial consortium

During decolourisation experiment, the optical density was continuously measured till 192 h at 12 h periodic interval by taking absorbance at 620 nm by UV–Vis spectrophotometer as the measurement of the bacterial cell growth. While the bacterial sample for biomass determination and SEM micrograph was collected from the flask after 144 h of incubation. First, the bacterial cells were centrifuged (6500×*g*) for 10 min at 4 °C, the pellets were washed thrice with distilled water to remove the attached medium contents. For bacterial biomass determination, the pellet was dried in an hot air oven at 80 °C until getting a constant dried weight reported in the form of dry cell mass (g L^−1^). For SEM micrograph, the bacterial cells were fixed with 1% (w/v) glutaraldehyde in 0.1 M phosphate buffer (pH 7.2) for 2 h and washed again with distilled water. The fixed samples were then dehydrated through 25, 50, 75, 95 and 100% ethanol solutions in increasing concentration for 5 min, at each step. The samples were then dried in critical point drier and coated with a thin conductive film of gold in a sputtering coater and observed under SEM (FEI Quanta 450, Hillsboro, USA).

### Estimation of MnP and lacasse activity

To measure the ligninolytic activity during degradation, the bacterial degraded supernatant was obtained by centrifugation at 6500×*g* for 10 min at 4 °C. The MnP and laccase activity was determined using the phenol red (Lobachemie, Mumbai, India) and guaiacol (Sigma-Aldrich, St. Louis, MO, USA), respectively as described earlier (Arora et al. 2002). The enzyme activity was expressed as international unit (IU), where 1 IU represents the amount of enzyme that forms 1 µmol of product per minute under standard assay conditions.

### UV–Vis and FT-IR spectroscopic analysis

UV–Vis absorption spectra of the treated and untreated SGA-MRPs solution were recorded using a UV–Vis spectrophotometer in the wavelength range between 200 and 700 nm at room temperature. Further, FT-IR analysis of the sample was performed in range of 400–4000 cm^−1^ using a spectrophotometer (Nicolet™ 6700, Thermo Scientific, USA) in order to reveal the chemical nature of the SGA-MRPs. The separated samples of SGA-MPs were mixed with potassium bromide (KBr) to prepare the pellet for FT-IR analysis.

### Extraction and identification of various organic compounds

The organic compounds present in treated and untreated SGA-MPs were extracted by ethyl acetate under acidic condition (pH < 2.0) as previously described (Chandra and Kumar 2017). An aliquot of the concentrate was dissolved in 3.0 mL methanol, filtered through 0.22-µm syringe filters and used for further HPLC and GC–MS analysis.

#### HPLC analysis

HPLC analysis was carried out using waters, 515 HPLC instrument equipped with a diode array detector system (1100 series, Agilent Technologies, USA) and reverse phase C_18_ column (250 × 4.6 mm, 5 µm particle size) by using the gradient of solvent A (Milli-Q water) and solvent B (acetonitrile with 0.1% TFA) (Merck, Germany) at a flow rate 0.4 mL min^−1^ for 60. The detection was monitored at wavelength 250 nm (absorption maxima) to assess the decolourisation and degradation of Maillard products. 50 µL of methanol extract was injected into the column by using a glass microsyringe (Agilent Technologies, USA).

#### GC–MS analysis

In GC–MS analysis, the extracted samples were derivatised with trimethylsilyl (TMS) as described by Chandra and Kumar (2017). An aliquot (2.0 µL) of derivatised sample was injected in GC–MS (Trace GC Ultra Gas Chromatograph, Thermo Scientific, USA) equipped with a TriPlus auto sampler coupled to TSQ Quantum XLS triple quadrupole mass spectrometer (Thermo Scientific, USA). Separation was carried out on DB-5 MS capillary column (30-m length × 0.25 µm I.D. × 0.25 mm film thickness of 5% phenyl and 95% methylpolysiloxane) with helium as the carrier gas at a flow rate of 1.1 mL min^−1^. The temperature of GC oven was programmed started from 65 °C (hold for 2 min), increased to 230 °C at a rate of 6 °C min^−1^ and finally reached to 290 °C (hold for 20 min) at a rate of 10 °C min^−1^ increased. The transfer line temperature and ion source temperature were kept at 290 and 220 °C, respectively. The mass spectrum (MS) was operated in the positive electron ionization (+ EI) mode at an electron energy of 70 eV with a solvent delay of 7 min. The MS was operated in full-scan mode from *m/z*, 45–800. The detected organic compounds were identified by matching with the MS library NIST v. 1.0.0.12 available with instrument.

### Toxicity assessment of Maillard reaction products

The toxicity effect of untreated and treated SGA-MRPs was studied on *P. mungo* L. seed germination using Petri dish method (Chandra and Kumar 2017). The seed germination parameters like germination percentage, germination index (GI), relative toxicity percentage, phytotoxicity percentage and stress tolerance index were calculated using the formula described earlier (Chandra and Kumar 2017; David Noel and Rajan 2015).

### Statistical data analysis

All the experiment was carried out in triplicate and the results were presented as the mean of three independent observations. To confirm the variability of data obtained and validity of results, the mean concentration of various physico-chemical parameters of untreated and treated SGA-MRPs was calculated by Student’s *t* test by using SPSS (version 17.0, Chicago, USA).

## Results

### Isolation and screening of bacterial strains

Twenty-seven morphologically distinct bacterial strains were isolated from distillery sludge. Only four bacterial isolates namely IITRCS01, IITRCS06, IITRCS07, and IITRCS11 could show the MnP activity by changing the deep orange to light yellow color at melanoidins (800 mg L^−1^) containing phenol red amended modified GPM agar plates, as well as laccase activity by developing a reddish brown color zone on B&K agar plate during the screening of potential bacterial strains capable for growth on SGA-MRPs amended media. The screened bacterial strains also showed tolerance for growth on melanoidins amended GPM agar plate at 3500 mg L^−1^ concentration.

### Characterisation and identification of bacterial strains

Screened bacterial strains IITRCS01, IITRCS06, IITRCS07, and IITRCS11 showed several morphological and biochemical characteristics are mentioned in Bergey’s manual of Systematic Bacteriology (Brenner et al. 2005). Further, the molecular identification of screened bacterial strains IITRCS01, IITRCS06, IITRCS07, and IITRCS11 showed specific amplification of a single band of around 1500 bp of 16S rRNA gene (Supplementary Fig. S1). The BLAST analysis of nucleotide sequence data of representative isolates was showed 100% sequence homology with *Klebsiella pneumoniae* (KU726952), *Salmonella enterica* (NR044372), *Enterobacter aerogenes* (NR024643), and *Enterobacter cloaceae* (KJ437492) found in the GenBank database. Hence, based on the sequence homology, the bacterial strain IITRCS01, IITRCS06, IITRCS07, and IITRCS11 were identified as *K. pneumoniae* (KU726953), *S. enterica* (KU726954), *E. aerogenes* (KU726955), *E. cloaceae* (KU726957), respectively. These bacterial species belonged to group *Gammaproteobacteria*. Further, the phylogenetic tree was showed the interrelationship of bacterial species with the most closely related genera inferred from sequences of the 16S rRNA gene as shown in Supplementary Fig. S2.

### Physico-chemical analysis

The physico-chemical characteristics of untreated SGA-MRPs showed dark brown in color at pH 6.9. In addition, the concentration of BOD (8641), COD (11,586), TDS (1050.77), VS (457.83), and phenolics (110.56 mg L^−1^) were found higher as shown in Table 1. In contrast to above, the bacterial treated SGA-MRPs showed reduction in all physico-chemical parameters as shown in Table 1. The colour of SGA-MRPs is dark brown which turned light brown after bacteria treatment. Initially, during degradation of SGA-MRPs the pH of medium was decreased to 4.1, but after 192 h of bacterial growth the pH was gradually enhanced. However, a sharp reduction in BOD (2568), COD (4201), TDS (687.92), VS (113.41) and phenolics (55.77 mg L^−1^) was noted as shown in Table 1. All the physico-chemical parameters of treated SGA-MRPs were found significantly higher (p < 0.001) that the untreated SGA-MRPs except for pH (p < 0.05).

**Table 1:** Physico-chemical analysis of sucrose-glutamic acid Maillard products

Parameter	Untreated (control)	Bacterial treated		Reduction (%)	Discharge permissible limit (USEPA 2002)
		After 96 h	After 192 h		
Color appearance	Dark brown	Dark brown	Light brown	–	–
pH	6.9 ± 0.0	4.1 ± 0.10^b^	8.1 ± 0.20^a^	− 14.28	–
BOD	8641 ± 160.33	5472 ± 1.04^a^	2568 ± 2.01^a^	70.28	40.00
COD	11586 ± 92.82	4867 ± 1.29^a^	4201 ± 3.11^a^	63.74	120.00
TDS	1050.77 ± 2.39	997.1 ± 1.01^b^	687.92 ± 2.90^a^	34.53	–
VS	457.83 ± 9.39	254.24 ± 1.11^a^	113.41 ± 1.12^a^	75.22	–
Phenolics	110.56 ± 1.40	89.01 ± 0.01^b^	55.77 ± 1.43^a^	49.55	750.00

All values are mean (n = 3) ± SD in mg L^−1^ except pH

*BOD* biological oxygen demand, *COD* chemical oxygen demand, *TDS* total dissolved solid, *VS* volatile solids

Student’s *t* test (two tailed as compared with untreated sample) was performed, where

^a^Highly significant at p < 0.001

^b^Significant at p < 0.01

### Effect of different carbon and nitrogen sources

**Fig. 1:**
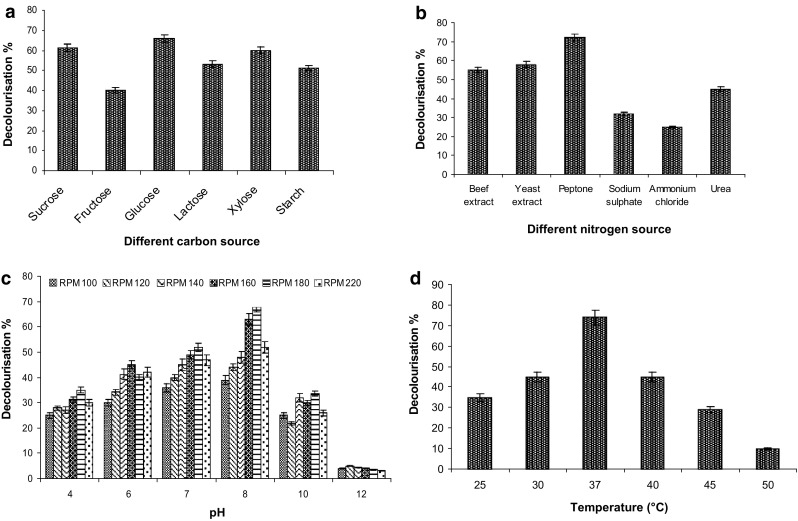
Effect of different nutrient and environmental parameters on SGA-MRPs decolourisation. **a** Carbon source, **b** nitrogen source, **c** pH, and **d** temperature and shaking speed at 37 °C for 24–192 h incubation

SGA-MRPs decolourisation was monitored by using different carbon sources at 1% (w/v) concentration for 192 h of incubation, and the results are illustrated in Fig. 1a. It was observed that glucose was the best carbon source, allowing maximum decolourisation (65.8%) at 1% (w/v) concentration. The decolourising ability of bacterial consortium was tested at different concentration showed that the efficiency of consortium increase with increase in glucose concentration from 0.1 to 1% reaching maximum decolourisation (65.8%) at 1.0%. While in absence of glucose slow growth of bacterial consortium was noted. However, sucrose, xylose and lactose were found less effective than glucose showed decolourisation upto 63–61.2%. Fructose was relatively poor co-substrate, aiding only 40% decolourisation.

The effect of different organic and inorganic nitrogen sources at a concentration of 0.5% (w/v) along with glucose (1.0% w/v) showed that peptone was most effective organic nitrogen source which enhanced up to 72% decolourisation of Maillard products at 0.1% (w/v) concentration (Fig. 1b). Further, increase in concentration of peptone resulted in continuous reduction in degradation capability of the developed bacterial consortium inhibited the decolourisation process. However, presence of other organic nitrogen sources i.e. beef extract, and yeast extract could show the SGA-MRPs decolorization upto 55–58% only, while urea could shown the decolourisation upto 15%, whereas inorganic nitrogen sources such as sodium sulphate and ammonium chloride could show the decolourisation upto 32, and 25%, respectively.

### Effect of pH, temperature and shaking speed

The effect of variable pH, temperature, shaking speed on Maillard products decolourisation showed that alkaline pH (8.1) was optimum for maximum solubility of Maillard products. Further, increase in pH inhibited the decolourisation process (Fig. 1c). Similarly, it was also observed that increase in temperature (25–37 °C) enhanced decolourisation of MRPs from 35 to 70% (Fig. 1d). While further, the increase in temperature up to 45 °C adversely affected the growth and decolourisation ability of the bacterial consortium. Further, the shaking speed effect from 100 to 180 rpm showed the optimum decolourisation by potential bacterial consortium at 180 rpm as shown in Fig. 1c. The bacterial consortium showed better decolourisation and degradation of SGA-MRPs in ration of 1:1:1:1.

### Bacterial growth and biomass

**Fig. 2:**
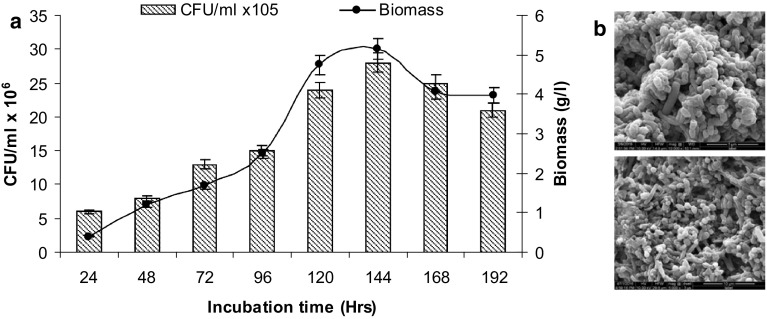
**a** Growth pattern and **b** scanning electron micrograph of developed bacterial consortium during degradation and decolourisation of SGA-MRPs

The periodic monitoring of bacterial growth curve and biomass showed maximum OD_620_ (2.49) and biomass (5.15 g L^−1^; CFU/mL 28 × 10^6^) at 144 h incubation period during decolourisation process (Fig. 2; Supplementary Fig. S3). This indicated the most optimum condition for maximum bacterial growth and MRPs decolourisation (74%). Beside, the SEM analysis of bacterial biomass showed thick biomass under SEM study also supported the high biomass of bacteria.

### Enzyme activity

To observe the function of ligninolytic enzymes in the decolourisation and degradation of SGA-MRPs, enzymes were measured and MnP was found to be the dominating enzymes at the initial stage of decolourisation. MnP activity was noted maximum at 120 h (2.8 U mL^−1^ min^−1^), and laccase activity was found maximum (1.91 U mL^−1^ min^−1^) at 144 h of incubation (Supplementary Fig. S4). But, after 120 and 144 h incubation showed gradual decrease of MnP and laccase enzyme activities of bacterial consortium, respectively. The colour reduction of MRPs was significantly initiated at 72 h and achieve maximum decolourisation at 144 h incubation.

### UV–Vis and FT-IR spectroscopy

**Fig. 3:**
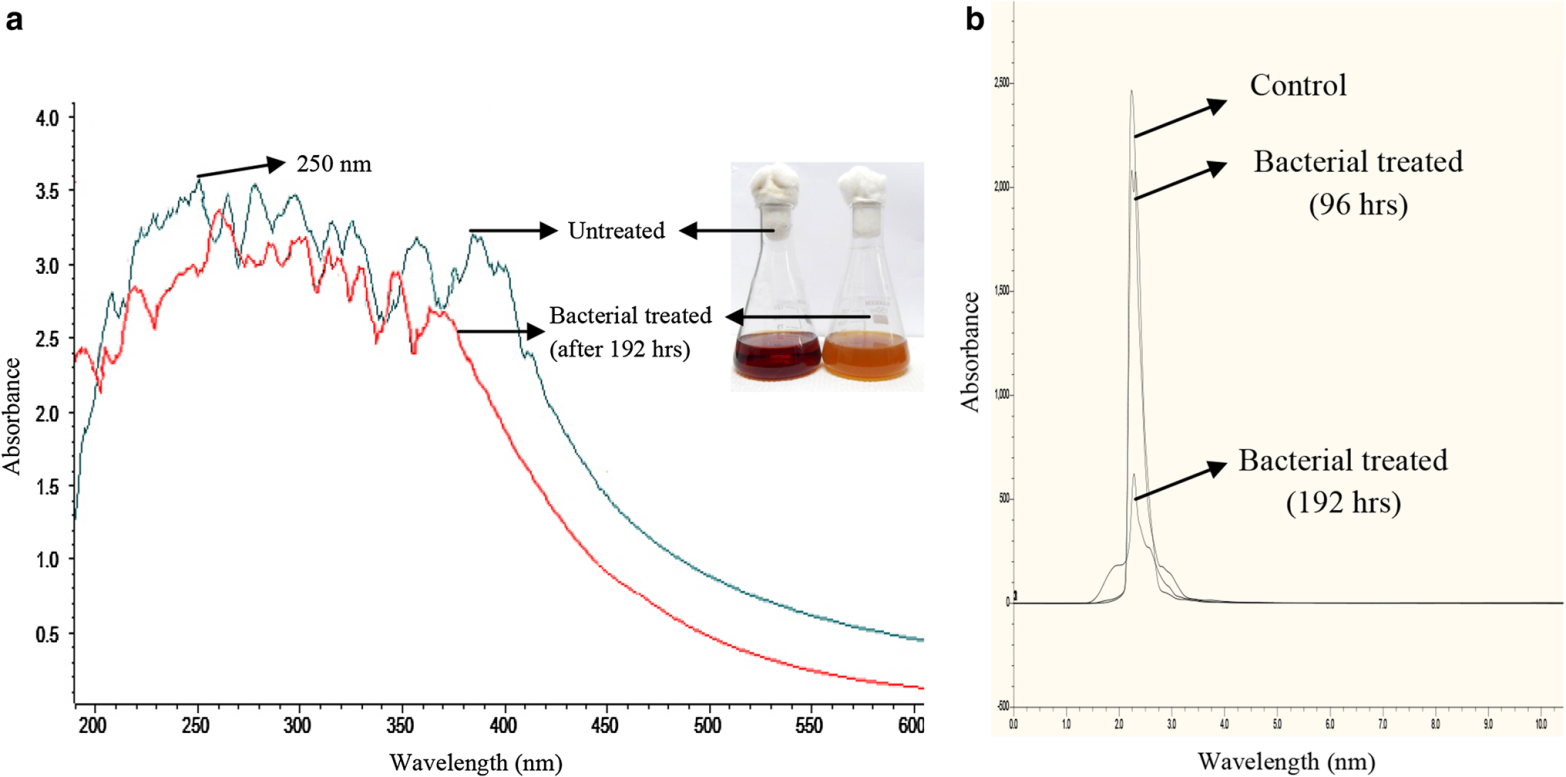
Analysis of untreated and treated SGA-MRPs at different time interval by **a** UV–Vis spectrum, and **b** HPLC analysis (at 250 nm)

The UV–Vis spectroscopy analysis in range between 200 and 700 nm for the change of Maillard products was done at different time intervals during the optimum decolourisation of SGA-MRPs. The result revealed that Maillard products showed different absorption peaks in the UV region and their maximum absorbance was noted at 250 nm (Fig. 3a). In addition, MRPs showed different stable peaks in a range of between 200 and 410 nm (Fig. 3a). The peaks were decreases as the day progresses, which showed the decolourisation of MRPs by bacterial consortium at the end of the 192 h of incubation.

FT-IR analysis supported the depolymerisation of SGA-MRPs after bacterial treatment by shifting the various functional groups as shown in Supplementary Fig. S5. The FT-IR spectrum of the SGA-MRPs is characterized by an intense absorption between 3200 and 3600 cm^−1^ represented the broad peaks of stretching vibration of O–H of COOH and the N–H stretching of amides. The region between 2800 and 3000 cm^−1^ exhibited the stretching of C–H from –CH_3_ present in hydrocarbons chain. The control sample showed the peaks at 2976.8, and 2930.3 cm^−1^ of C–H asymmetric stretching vibration of –CH, and –CH_3_ functional groups. The peak value 2930.3, and 2946.8 was represented the C–H vibrational stretching from CH_3_ present in hydrocarbon chain. An intense band in the 1500–1750 cm^−1^ range (1596.0 and 1739.5) may correspond to carbonyl groups in primary amide functions, or it could appear because of the existence of C–O stretching of COO^−^ ketonic C–O and aromatic C–C conjugated with COO^−^. The absorption band at 1411.1 and 1457.8 cm^−1^ might appear due to aliphatic C–H bending and COO^−^ asymmetric stretching in spectra. The untreated sample showed absorptions peak between 1000 and 1200 cm^−1^ in untreated and simulated spectra could appear due to C–O bonds (ethers and alcohols) and/or minerals. While a band at 922.8, and 978.0 cm^−1^ indicated the presence of C–O stretching of polysaccharides or Si–O-asymmetric stretch. The untreated sample also showed an intense peak at 847.7, 778.5, 629.6 and 535.4 cm^−1^ represent the sulfate, carbohydrate, alkyl halides, nitro group, and C–S bond. The treated sample showed a narrow peak at 3414.0 cm^−1^ represented the stretching vibration of O–H group. The spectra corresponding to treated sample was displays bands at 2855.4 and 2924.8 cm^−1^, probably due to stretching vibrations of CH_3_ and CH_2_ groups, respectively. The broad stretching adsorption band peak at 2160.0 cm^−1^ could be assigned to C=C stretching vibrations. The absorption band at 1444.9 cm^−1^ might appear due to aliphatic C–H bending. The occurrence of aromatic groups is also suggested by absorptions at around 700 cm^−1^. Moreover, most of the bands were similar between untreated and simulated samples.

### HPLC analysis

The HPLC analysis report demonstrating the area, height, and retention time (RT), before and after bacterial treatment of Maillard products which confirms the degradation of melanoidins (Fig. 3b). The HPLC analysis of untreated samples extracted with ethyl acetate showed a single major peak at RT of 2.40 min, while in 96 h treated sample was less compared to untreated sample clearly indicates the capability of the bacterial consortium to decolorize and degrade the SGA-MRPs by their enzymatic action. In addition, there were some extra peaks appeared in bacterial treated sample suggesting the formation of several new metabolites. Further, after 192 h treatment of SGA-MRPs, the HPLC chromatogram also showed shifting of peak, decrease in peak area height, and generation of some new peaks compared to untreated sample. This suggested the biotransformation and biodegradation of SGA-MRPs in various metabolites by developed bacterial consortium.

### Identification of organic pollutants and their metabolites

**Fig. 4:**
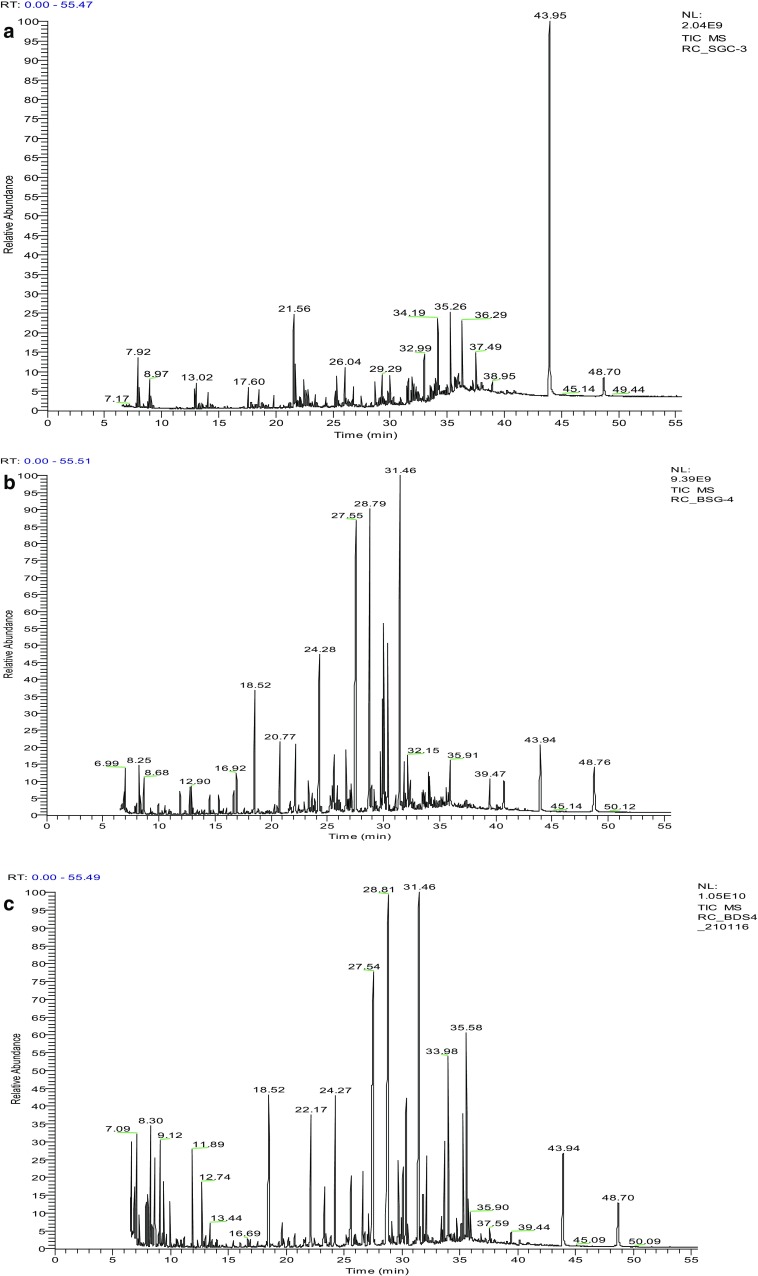
GC–MS chromatogram of organic compounds extracted with ethyl acetate from SGA-MRPs. **a** Control, **b** bacterial degraded after 96 h incubation, and **c** bacterial degraded after 192 h incubation

In the present study, the GC–MS analysis of ethyl acetate extracted untreated SGA-MRPs showed the existence of different types of organic pollutants, most of which were biotransformed and biodegraded during bacterial treatment (Fig. 4; Table 2). The major organic compounds detected in untreated sample at different RT were nonane, 3,7-dimethyl (RT:7.76), 1-octanol,2,2-dimethyl (7.85), dodecane, 2,6, 10-trimethyl (RT:8.05), d-lactic acid-DiTMS (RT:8.34), 1-dodecene (RT:8.41), undecane, 2-methyl (RT:8.80), butanoic acid, TMS ester (RT:9.12), dodecane (RT:9.28), silanol, trimethyl, benzoate (RT:12.36), heptadecane, 2,6,10,15-tetramethyl (RT:13.22), benzeneacetic acid, TMS ester (RT:13.44), 1-tetradecanol (RT:13.60), 2-bromo dodecane (RT:13.93), pentadecane (RT:14.03), dodecane, 2,6,11-trimethyl (RT:14.07), heptadecane (RT:14.47), tetradecane (15.77), hexadecane, 2,6,10,14-tetramethyl (RT:17.60), phenol,2,4-bis (1,1dimethylethyl) (RT:17.95), 1-tetradecanol (RT:18.17), tetradecane, 2,6,10-trimethyl (RT:18.30), hexadecane (RT:19.82), dodecanoic acid, TMS ester (RT:20.77), docosane (RT:21.68), pyrazine, 2,5-dimethyl-3-propyl (RT:21.56), 2,6-diisopropylnaphthalene (RT:22.19), 3,4-tetramethylene-5,5-pentamethyle-2-nepyrazoline (RT:22.66), Hexadecane, 2,6,11,15-tetramethyl (RT:25.64), 7,9-di-*tert*-butyl-1-oxaspiro(4,5)deca-6,9-diene-2,8-dione (RT:25.21), heptacosane (RT:29.40), hexadecanoic acid, TMS ester (RT:27.55), 1H-purin-6-amine,[(2-fluorophenyl)]methyl (RT:27.90), pentacosane (RT:29.29), 2,2-bis [(4-trimethylsiloxy)phenyl]propane (RT:30.00), nonacosane (RT:32.30), 1,2-benzenedicarboxylic acid, bis (2-ethylhexyl)ester (RT:33.51), hexadecanoic acid (RT:33.67), octadecane, 3-ethyl-5-(2-ethylbutyl) (RT:33.97), and methylenebis (2,4,6-triisopropylphenylphosphine) (RT:43.94). However, the investigation of bacteria treated SGA-MPs sample has showed the existence of various organic compounds such as, 3-hydroxy-2-butanone, TMS ester (RT:9.41), ethanolamine, TBS (RT:9.65), butane, 2,3-bis (TMSoxy) (RT:10.52), silanol, trimethyl, pentadecane (RT:14.03), undecenoic aicd, TMS ester (RT:18.51), *cis*-5-dodecanoic acid, TMS ester (RT:22.71), tetradecanoic acid, ethyl ester (RT:24.28), 2-hexadecanol acetate (RT:25.52), *trans*-9-octadecenoic acid 1TMS (25.60), *cis*-10-heptadecenoic acid, TMS ester (RT:28.79), octadecanoic acid, ethyl ester (RT:29.72), 11-*trans*-octadecenoic acid, TMS ester (RT:30.10), methyl 19-methyl-eicosanoate (RT:32.16), 1-docosanol, acetate (RT:33.40), docosanoic acid, methyl ester (34.08), 17-pentatriacontene (RT:35.13), 2-monostearin, TMS ether (RT:35.30), octadecanoic acid, 2,3-bis [(TMS)oxy]propyl ester (RT:35.58), and ethyl tetracosanoate (RT:35.72) at different RT. These compound were completely absent in untreated sample, they are the degraded products of SGA-MRPs and few as new metabolites. In bacterial treated sample two organic compounds i.e. docosane (RT:21.68) and dodecanoic acid, TMS ester (RT:20.77) could not be metabolized.

**Table 2:** Organic compounds identified by GC–MS analysis extracted with ethyl acetate from untreated (control) and bacterial treated SGA-MRPs

SI. No.	Name of compound	RT	C	BT	
				96 h	192 h
1	Nonane, 3,7-dimethyl	7.76	+	−	−
2	Butane, 2,3-bis(trimethylsiloxy)	7.82	−	+	−
3	1-Octanol,2,2-dimethyl	7.85	+	−	−
4	3,6-Dioxa-2,7-disilaoctane,2,2,4,5,7,7-hexamethyl	8.00	−	+	−
5	Dodecane, 2,6, 10-trimethyl	8.05	+	−	−
6	d-Lactic acid-DiTMS	8.34	+	−	−
7	1-Dodecene	8.41	+	−	−
8	Piperidine, 1,4-dimethyl	8.68	−	+	−
9	Undecane, 2-methyl	8.80	+	−	−
10	Butanoic acid, TMS ester	9.12	+	−	−
11	Dodecane	9.28	+	+	−
12	3-Hydroxy-2-butanone. TMS ester	9.41	−	−	+
13	Ethanolamine, TBS	9.65	−	−	+
14	Silane, trimethyl(4-methylphenoxy)	10.31	−	+	−
15	Butane, 2,3-bis(TMSoxy)	10.52	−	−	+
16	α-d-Mannopyranoside, methyl, cyclic 2,3:4,6-bis(butyloboronate)	10.53	−	+	−
17	2-Hexenoic acid, 5-(1-ethoxyethoxy), 2-(TMS)ethyl ester	10.57	−	+	−
18	Silanol, trimethyl, benzoate	12.36	+	−	−
19	Octanoic acid, TMS ester	12.75	−	+	−
20	Silanol, trimethyl-phosphate	12.90	+	−	−
21	Heptadecane, 2,6,10,15-tetramethyl	13.22	+	−	−
22	Benzeneacetic acid, TMS ester	13.44	+	−	−
23	1-Tetradecanol	13.60	+	−	−
24	Butanedioic acid, bis(TMS)ester	13.82	−	+	−
25	2-Bromo dodecane	13.93	+	−	−
26	Pentadecane	14.03	+	−	+
27	Dodecane, 2,6,11-trimethyl	14.07	+	−	−
28	Vanillin, *tert*-butyldimethylsilyl ether	14.45	−	+	−
29	Heptadecane	14.47	+	−	−
30	4-Methoxy-α-(TMS)benzenemethanol	14.51	−	+	−
31	Nonanoic acid, TMS ester	14.85	−	+	−
32	1H-Purine-2,6-dione, 3,7-dihydro-1,3-dimethyl-7-(TMS)	15.34	−	+	−
33	Tetradecane	15.77	+	−	−
34	Decanoic acid, TMS ester	16.92	−	+	−
35	Hexadecane, 2,6,10,14-tetramethyl	17.60	+	−	−
36	2-(2-(2-Butoxyethoxy)ethoxy)ethoxy-trimethylsilane	17.84	+	−	−
37	Phenol,2,4-bis(1,1dimethylethyl)	17.95	+	−	−
38	1-Tetradecanol	18.17	+	−	−
39	Tetradecane, 2,6,10-trimethyl	18.30	+	−	−
40	Undecenoic aicd, TMS ester	18.51	−	−	+
41	Undecenoic acid, TMS ester	18.52	−	+	−
42	Hexadecane	19.82	+	−	−
43	Dodecanoic acid, TMS ester	20.77	+	+	+
44	Pumiliotoxin	20.52	−	+	−
45	Docosane	21.68	+	+	+
46	Pyrazine, 2,5-dimethyl-3-propyl	21.56	+	−	−
47	Glucopyranose-1,2,3,5-Di-methylboronate-TMS	22.11	−	+	−
48	2,6-Diisopropylnaphthalene	22.19	+	−	−
49	3,4-Tetramethylene-5,5-pentamethyle-2-nepyrazoline	22.66	+	−	−
50	*Cis*-5-Dodecanoic acid, TMS ester	22.71	−	+	+
51	Leucine	22.82	+	−	−
52	Tetradecanoic acid, ethyl ester	23.32	−	+	−
53	Myristoleic acid ITMS	23.87	−	+	−
54	Tetradecanoic acid, TMS ester	24.20	−	+	−
55	Tetradecanoic acid, ethyl ester	24.28	−	−	+
56	Hexadecane, 2,6,11,15-tetramethyl	25.34	+	−	−
57	7,9-Di-*tert*-butyl-1-oxaspiro(4,5)deca-6,9-diene-2,8-dione	25.21	+	−	−
58	n-Pentadecanoic acid, TMS ester	25.43	−	+	
59	2-Hexadecanol acetate	25.52	−	−	+
60	1,4-Diaza-2,5-dioxa-3-isobutyl bicycle nonane	25.60	−	+	−
61	*Trans*-9-Octadecenoic acid 1TMS	25.60	−	−	+
62	Heptacosane	29.40	+	−	−
63	Hexadecanoic acid, TMS ester	27.55	+	+	+
64	1H-Purin-6-amine,[(2-fluorophenyl)]methyl	27.90	+	−	−
65	*Cis*-9-Hexadecenoic acid, TMS ester	27.14	−	+	−
66	*Cis*-10-Heptadecenoic acid, TMS ester	28.79	−	+	+
67	Pentacosane	29.29	+	−	−
68	Octadecanoic acid, ethyl ester	29.72	−	+	+
69	9,12-Octadecadienoic acid, TMS ester	29.93	−	+	−
70	2,2-Bis[(4-trimethylsiloxy)phenyl]propane	30.00	+	−	−
71	11-*Trans*-Octadecenoic acid, TMS ester	30.10	−	+	+
72	Octadecanoic acid, TMS ester	30.38	−	+	−
73	*Cis*-10-Nonadecenoic acid, TMS ester	31.46	−	+	+
74	Methyl 19-methyl-eicosanoate	32.16	−	+	+
75	Nonacosane	32.30	+	−	−
76	2-Acetyl-3-(2-cinnamido)ethyl-7-methoxyindole	32.38	−	+	−
77	17-Pentatriacontane	33.41	−	+	−
78	1,2-Benzenedicarboxylic acid, bis(2-ethylhexyl)ester	33.51	+	−	−
79	1-Docosanol, acetate	33.40	−	−	+
80	Hexadecanoic acid	33.67	+	−	−
81	Dotriacontane	33.90	−	+	−
82	Octadecane, 3-ethyl-5-(2-ethylbutyl)	33.97	+	−	−
83	Hexadecanoic acid, 2,3-bis[(TMS)oxy]propyl ester	33.98	−	−	+
84	Hexadecanoic acid, 2,3-bis[(TMS)oxy]propyl ester	33.99	−	+	−
85	Docosanoic acid, methyl ester	34.08	−	−	+
86	Ethyl docosanoate	34.09	−	+	−
87	*Cis*-13-docodenoic acid, TMS ester	34.24	−	+	−
88	Docosanoic acid, TMS ester	34.45	−	+	−
89	Quercetin 7,3′,4′,-trimethoxy	34.72	−	+	−
90	Heptacosane	34.99	−	+	−
91	17-Pentatriacontene	35.13	−	−	+
92	2-Monostearin, TMS ether	35.30	−	+	+
93	Octadecanoic acid, 2,3-bis[(TMS)oxy]propyl ester	35.58	−	−	+
94	Ethyl tetacosanoate	35.72	−	−	+
95	2,6,10,14,18,22-Tetracohexane, 2,6,10,15,19,23-hexamethyl	35.91	−	+	−
96	Silane, [[(3β)-cholest-5-en-3-yl]oxy]TM	39.47	−	+	−
97	Silane, (ergosta-5,7,22-trien-3β-yl)TM	40.71	−	+	−
98	Methylenebis (2,4,6-triisopropylphenylphosphine)	43.94	+	−	−

*TMS* trimethylsilyl, *C* control, *BT* bacterial treated, *RT* retention time

### Effect of SGA-MRPs on seed germination and seedling growth

The phytotoxicity of untreated and treated sample was assessed by observing the germination percentage and measuring the length of the radical of *P. mungo* L. seeds. It was observed that untreated sample was highly toxic in nature and showed only 75, 70, 60, 15% germination at 2.5, 5, 10 and 15% concentration of SGA-MRPs. But, after treatment with bacterial, the toxicity of SGA-MRPs was reduced extensively and showed 90, 90, 80, 25, and 20% germination at 2.5, 5, 10, 15, and 20% concentration of Maillard products (Table 3; Supplementary Fig. S6). The percent germination and radical length were combined for a comprehensive interpretation of Maillard products toxicity in term of GI. The GI of untreated and treated SGA-MRPs values ranged between 0.05–0.61 and 0.10–0.85, respectively. The GI was decreased with increased concentration of treated sample compared to untreated sample. The percent phytotoxicity analysis of untreated sample revealed that the phytotoxicity increased with increased in SGA-MRPs concentration but in degraded SGA-MRPs it gradually decreased. Various parameters were studies showed seedling vigor index (16.5–180 and 30–252), stress tolerance index (37.28–81.35 and 50.84–94.91), relative toxicity (85–25 and 80–10) with untreated and treated SGA-MPs, respectively. These results clearly indicated that the toxicity of SGA-MRPs was reduced appreciably after bacterial treatment.

**Table 3:** Effect of untreated and bacterial treated SGA-MRPs on the seed germination and seedling growth of *Phaseolus mungo* L

Samples	Treatment (%)	Germination (%)	Germination index	Phytotoxicity (%)	Seedling vigor index	Stress tolerance index (%)	Relative toxicity (%)	Radical length (cm)
Untreated	2.5	75 ± 0.00	0.61 ± 0.00	18.64 ± 0.09	180 ± 8.01	81.35 ± 0.02	25 ± 0.01	2.40 ± 0.01
	5	70 ± 0.00	0.48 ± 0.00	30.50 ± 0.06	143.50 ± 4.21	69.49 ± 0.02	30 ± 0.03	2.05 ± 0.01
	10	60 ± 0.00	0.30 ± 0.00	49.15 ± 0.07	90 ± 0.08	50.84 ± 0.01	40 ± 0.02	1.50 ± 0.00
	15	15 ± 0.00	0.05 ± 0.00	62.71 ± 0.04	16.5 ± 0.01	37.28 ± 0.01	85 ± 0.11	1.10 ± 0.00
	20	NG	–	–	–	–	–	–
Treated	2.5	90 ± 0.00	0.85 ± 0.00	5.08 ± 0.01	252 ± 14.01	94.91 ± 0.03	10 ± 0.00	2.80 ± 0.01
	5	90 ± 0.00	0.71 ± 0.00	20.33 ± 0.01	211.5 ± 9.14	79.66 ± 0.02	10 ± 0.00	2.35 ± 0.01
	10	80 ± 0.00	0.61 ± 0.00	23.05 ± 0.03	181.6 ± 4.21	76.94 ± 0.02	20 ± 0.01	2.27 ± 0.02
	15	25 ± 0.00	0.18 ± 0.00	27.11 ± 0.02	53.75 ± 0.04	72.88 ± 0.01	75 ± 0.09	2.15 ± 0.01
	20	20 ± 0.00	0.10 ± 0.00	49.15 ± 0.01	30 ± 0.00	50.84 ± 0.01	80 ± 0.17	1.50 ± 0.00
Control	0	100 ± 0.00	–	–	295 ± 24.01	–	–	2.95 ± 0.01

All values are mean of three replicates ± SD

*NG* no germination, *control* tap water

## Discussion

In the present study, bacterial isolates IITRCS01, IITRCS06, IITRCS07, and IITRCS11 showed prominent MnP and laccase activity during the screening on GPM agar plates amended with phenol red and B&K agar plate amended with guaiacol. The phenol red changed from deep orange to light yellow color during screening of peroxidase activity in bacteria has been used as an indicator of MnP activity shown in Supplementary Fig. S4. This change in colour of phenol red occurred due to oxidation of glucose by sugar oxidase enzyme, resulting in the production of H_2_O_2_ and media acidification (i.e. lowering of pH) which is required for the melanoidins degradation (Aoshima et al. 1985; Hwang et al. 2011). The guaiacol is also a chromogenic substrate that is used for quick screening of microbial strains producing laccases by means of a color reaction (Okino et al. 2000). The selected four bacterial strains produced the laccase which catalyzed the oxidation of guaiacol to form reddish brown halo zones in the medium (Supplementary Fig. S4). This observation corroborated with earlier finding for screening of MnP and laccase producing bacterial strains by Chandra and Singh (2012).The 16S rRNA genes sequence analysis showed that screened bacterial strain IITRCS01, IITRCS06, IITRCS07, and IITRCS11 were identified as *K. pneumoniae* (KU726953), *S. enterica* (KU726954), *E. aerogenes* (KU726955), *E. cloaceae* (KU726957), respectively. This bacterial species belonged to group *Gammaproteobacteria*. The class *Gammaproteobacteria* constitutes a very large and diverse group of bacteria that exhibits enormous variety in terms of their phenotype and metabolic capabilities (Woese et al. 1985; Brenner et al. 2005; Kersters et al. 2006). Members of this group exhibit broad ranges of aerobicity, of trophism, including chemoautotrophism and photoautotrophism, and of temperature adaptation. The class *Gammaproteobacteri*a also includes enteric bacteria and it is well known for harbouring large numbers of human, animal and plant pathogens (Brenner et al. 2005; Kersters et al. 2006). Although, *Gammaproteobacteria* has only the taxonomic rank of class within the phylum *Proteobacteri*a.

Since, MR is a complex series of chemical reaction that occurs between the carbonyl groups of reducing sugars and the amino groups of amino acids, peptides or proteins (Echavarría et al. 2013a, b). The MRPs are a particularly complex mixture of various organic compounds of different molecular weights. They include not only aldehydes, ketones, dicarbonyls, acryl amides, and heterocyclic amines, but also melanoidins, which are polymeric products formed at the advanced steps of MR (Wang et al. 2011). In this study, we have selected sucrose, glutamic acid, and sodium carbonate for the synthesis of MRPs due to presence of sucrose and glutamic acid abundantly present in sugarcane juice, which are highly reactive in the MR at elevated temperature, sucrose invariably hydrolyses first to the reducing thermolabile hexose sugar, glucose and fructose, which enter into the initiation pathway of MR (Walford 1996; Nolasco Junior and De Massaguer 2006). The alkaline pH (8.0) favour the MR, because at alkaline pH sugar thermal degradation rate increased which leads formation of colour. Therefore, color formation under alkaline condition is result of sucrose degradation into reducing sugar at initial stage (Eggleston and Vercellotti 2000). In addition, the used of sodium carbonate also favour the reaction of MRPs formation during the decomposition of sugar. Further, the physico-chemical characteristics of untreated MRPs showed dark brown in color at high BOD, COD, TDS, VS, and phenolics at pH 6.9. Thus, the high BOD and COD values of the MRPs solution are due to lack of oxygen molecule in MRPs during the synthesis of MRPs after dehydration process. Since, some caramelized sugar may be also generated during the MRPs synthesis. Therefore, high TDS of solution is found in this study. However, in contrary to control, the bacterial degraded sample showed reduction of BOD, COD, TDS, VS and phenolics values. This indicated the degradation of MRPs by inoculated bacterial consortium due to presence of ligninolytic enzyme activities. Consequently, the reduction in colour was noted due to depolymerisation of melanoidins as reported earlier (Verman et al. 1974). At the initial stage of bacterial incubation during the decolourisation process the pH of medium was noted 4.1. This might be due to generation of some organic acids with bacterial reaction in medium composition (Davídek et al. 2006). But, it gradually moves towards alkaline condition (pH 8.1) after 192 h of bacterial incubation. The increased pH of media might be due to depolymerisation of MRPs by enzyme activity of bacterial consortium. This induced the solubility and utilisation of melanoidins which favoured the mineralisation and co-metabolism of MRPs (Bharagava et al. 2009). These results also corroborated to the previous findings regarding the degradation of natural and synthetic melanoidins (Bharagava et al. 2009; Kumar and Chandra 2006; Yadav and Chandra 2012).

A potential consortium was developed from selected strains of bacteria for decolourisation of MRPs. It has been reported glucose is an essential carbon source for melanoidins decolourisation (Kumar and Chandra 2006). In our investigation, the potential bacterial consortium showed maximum decolourisation (65.8%) at 1.0% concentration of glucose. While in absence of glucose the slow growth of bacterial consortium was noted. This revealed that the carbon source present in MRPs is not readily being utilised by bacterial consortium. But, the supplementation of glucose in media was readily available for bacterial growth. This indicated strong evidence that incubated bacterial consortium is not able to utilise the SGA-MRPs for its growth and metabolism. Similar observation has also been reported by previous worker in case of anaerobically treated distillery spent wash (Ghosh et al. 2004). This indicated that our bacterial consortium was more effective for decolourisation and degradation of MRPs. This might be due to induction of hight amount of ligninolytic enzyme which facilitated for the decolourisation processes (Kumar and Chandra 2006). Simultaneously, the effect of various concentration of organic (peptone, beef extract, yeast extract, and urea) and inorganic nitrogen (sodium sulphate and ammonium chloride) sources on MRPs decolourisation was investigated, but peptone was found most effective organic nitrogen source which showed decolourisation upto 72% at 0.1% (w/v) concentration. Because, peptone are rich in free amino acids and short peptides, which support cell growth with a low consumption rate of the carbon source and minimize the accumulation of by-products (Lau et al. 2004). Moreover, presence of other sources of organic nitrogen could show the decolorization upto 55–58% only, whereas inorganic nitrogen could show the decolourisation upto 15–32%. Thus, this finding showed the inhibitory effect of sodium sulphate and ammonium chloride on bacterial growth. Similar, effect has also been reported by previous workers for melanoidins decolourisation (Tiwari et al. 2012, 2013; Yadav and Chandra 2012; Sirianuntapiboon et al. 2004). Hence, the developed bacterial consortium utilised tiny amount of peptone for higher decolourisation, BOD, and COD reduction compared to other researchers ever reported.

The variation in SGA-MRPs decolourisation process was directly affected with change in pH and temperature of culture conditions. The optimum SGA-MRPs decolourisation (72%) was noted at pH 8.1 by developed bacterial consortium after 192 h of incubation. Further, increase or decrease in pH inhibited the decolourisation process. This might be due to inhibition of the enzyme activity. For developed aerobic bacterial consortium comprising *Bacillus* sp., *Bacillus licheniformis*, and *Alcaligenes* sp. was showed highest decolourisation activity at pH 7.0 (Bharagava and Chandra 2010). In another study, the optimum pH for melanoidins decolourisation by bacterial strains *Alcaligenes faecalis* SAG_5_ was note at pH 7.5 (Santal et al. 2011). The developed bacterial consortium showed maximum decolourisation (70%) at 37 °C. While further, increase in temperature up to 45 °C adversely affected the growth, metabolism and MRPs decolourisation ability of bacterial consortium. Similar pattern is also reported in other studies also (Mohana et al. 2007; Santal et al. 2011). However, the Maillard products were significantly affected by various shaking speed also. It was observed that the optimum decolourisation (70%) by potential bacterial consortium was noted at 180 rpm. However, increase in shaking speed resulted decrease in decolourisation process. This might be due to mechanical injury of bacterial cell wall at a higher shaking speed (Tiwari et al. 2014; Yadav and Chandra 2012).

For efficient and potential consortium, different inoculums ratio of bacterial strains were also optimized. Consortium having 1:1:1:1 inoculums ratio of bacteria showed maximum (74%) decolourisation of MRPs within biomass (5.15 g L^−1^; CFU mL^−1^ 28 × 10^6^) OD_620_ (2.49) at 144 h of incubation. Further, incubation did not increase the rate of decolourisation. The direct correlation of OD_620_ with high bacterial biomass indicated the direct involvement in decolourisation and degradation process of MRPs. This indicted high bacterial growth was due to utilisation of MRPs as a carbon source for its growth.

During the maximum decolourisation of MRPs, the MnP activity was noted highest (2.8 U mL^−1^ min^−1^) at 120 h of incubation and laccase activity was found highest (1.91 U mL^−1^ min^−1^) at 144 h of incubation. The MnP and laccase have a broad substrate oxidising enzymes reported for detoxification and degradation of phenolic and non-phenolic polymeric compounds (Rodriguez et al. 1999; González et al. 2008). The presence of melanoidins and other similar compounds can induce the expression of MnP and laccase protein both are having synergistic action in the degradation and depolymerisation of melanoidins and other industrial pollutants (Miyata et al. 2000; González et al. 2008). In our study, MnP was profusely present at the initial phase of bacterial growth, while laccase was produced in later phase of growth during MRPs degradation. The findings of this study are well supported by earlier results reported by various workers (Pant and Adholeya 2007, 2009b; Yadav and Chandra 2012).

UV–Vis spectrophotometric analysis is the most common used method of the MR (Martins and Van Boekel 2003; Echavarría et al. 2014). In this study, MRPs showed different absorption peaks in a range of between 200 and 410 nm and their maximum absorbance was noted at 250 nm (Fig. 3a). The stable peaks absorbance in UV region indicated the presence of several organic compounds formed during the early stages of the MR (Echavarría et al. 2013a, b). While, the absorbance of either 280 nm, 320–350 nm or 420–450 nm corresponding to early MRPs for pyrazine compounds (at 280 nm) (Gu et al. 2010), advanced stage for soluble pre-melanoidins formation (at 320–350 nm) and final MRPs (at 420–450 nm) corresponding to polymerised MRPs in medium (Billaud et al. 2004). On the basis of our findings, it can be stated that the formation of various peaks in UV and visible region indicated the presence of complex mixture of early, advanced and final MRPs. Our findings corroborated with the earlier results reported by Echavarría et al. (2013a, b).

The FT-IR spectrum of the SGA-MRPs is characterized by an intense absorption between 3200 and 3600 cm^−1^ represented the broad peaks of stretching vibration of OH of COOH and N–H of amides. The peak value 3519.4, 3486.2, 3427.6, 3397.9, 3366.1, and 3333.1 cm^−1^ was represented the O–H vibrational stretching present in acids, alcohol and phenol (Ramezani et al. 2011). The region between 2800 and 3000 cm^−1^ exhibited the stretching of C–H from –CH_3_ present in hydrocarbons chain (Kadam et al. 2013). The weakening of O–H group was probably due to action of bacterial enzyme which cleaved the C–O bond and eventually removed the O–H group from the polymer. These results are corroborated by previous findings (Liakos and Lazaridis 2014; Ramezani et al. 2011). The additional peaks in the treated effluent are due to degradation of SGA-MPs by developed bacterial consortium. In HPLC analysis, the SGA-MRPs showed major peak at 2.40 min but after bacterial treatment the height and area of peak reduced and some additional peaks was also observed. This indicated the biotransformation of Maillard products and formation of some new metabolites. These findings are well supported by earlier results reported by various researchers (Tiwari et al. 2012; Yadav and Chandra 2012).

GC–MS is an ideal technique for the determination of organic pollutants in environment. The major organic compounds detected in untreated sample were organic acids, pyrazine and phenolic compounds. Lactic acid, benzeneacetic acid, and benzenedicarboxylic acid are the alkaline degradation products of hexose. Pyrazines are the amadori products play a key role during the Maillard product synthesis. Phenolic compounds are the fragmentary product of amadori compound. The presence of organic acids, phenolics and long chain aliphatic compounds in wastewater was reported previously by various workers (Gonzalez et al. 2000; Chandra and Kumar 2017). However, the analysis of sample after bacteria treatment has showed the presence of various organic compounds as shown in Table 2. Similar, compounds have been identified in distillery effluent after treatment with *Emericella nidulans* var. lata, *N. intermedia* followed by *Bacillus* sp. in three stage bioreactor (Kaushik et al. 2010). However, several organic compounds detected in untreated sample were diminished after bacterial treatment. This suggested that complex and high molecular weight compounds were degraded by bacterial consortium with the help of extracellular ligninolytic enzymes. These organic compounds were utilized as sole carbon, nitrogen and energy source and played a key role in the decolourisation and degradation of SGA-MRPs.

The toxicity study of degraded and undegraded SGA-MRPs with *P. mungo* L. seeds showed that untreated sample was highly toxic in nature and showed only 75, 70, 60, 15% germination at different concentration of SGA-MRPs. While, the toxicity of degraded SGA-MRPs sample was significantly reduced. Suppression of germination at high concentration of SGA-MRPs might be due to the occurrence of highly toxic organic pollutants and dissolve solid which absorbed by the seed before germination and affecting different biochemical and physiological process of seed germination (Bharagava and Chandra 2010). Increased in percent germination in treated SGA-MRPs might be due to decrease of organic compounds that has created favorable environment for germination and utilisation of nutrient present in Maillard products.

## Conclusion

This study has revealed that SGA-MRPs showed mixture of variable molecular weight melanoidins. Therefore, several peaks were noted in rage of 200–450 nm. Further, this study also showed that the developed bacterial consortium has ability to degrade and decolourise recalcitrant complex SGA-MRPs polymer up to 70% in existence of adequate carbon and nitrogen source at broad range of temperature, pH, and shaking speed within a incubation period of 192 h. GC–MS and other spectrophotometric analysis of untreated and bacterial treated sample of SGA-MRPs have shown that the majority of the colours containing compounds (melanoidins) detected in untreated sample were diminished from the treated sample by MnP and laccase enzyme activity of bacteria. Hence, the optimized conditions for developed bacterial consortium can be used for decolourisation of MRPs containing industrial wastewater at large scale.

## Electronic supplementary material

Below is the link to the electronic supplementary material.

Supplementary material 1 (DOC 6094 KB)
