# Supplementary material for: Characterisation of manganese peroxidase and laccase producing bacteria capable for degradation of sucrose glutamic acid-Maillard reaction products at different nutritional and environmental conditions
Source: World J Microbiol Biotechnol. 2018 Feb 2;34(2):32. doi: 10.1007/s11274-018-2416-9 (PMC5797191; doi:10.1007/s11274-018-2416-9)
Supplement: Supplementary file 1 — Supplementary material 1 (DOC 6094 KB) [file 11274_2018_2416_MOESM1_ESM.doc]

**Supplementary Material**

**Characterisation of manganese peroxidase and laccase producing bacteria capable for degradation of sucrose glutamic acid-maillard products at different nutritional and environmental conditions**

**Vineet Kumar, Ram Chandra**

Department of Environmental Microbiology, School for Environmental Sciences, Babasaheb Bhimrao Ambedkar Central University, Vidya Vihar, Raebareli Road, Lucknow, Uttar Pradesh-226025, India

Corresponding author: [rc_microitrc@yahoo.co.in](mailto:rc_microitrc@yahoo.co.in); prof.chandrabbau@gmail.com

**Supplementary Figures**

**M**

**N**

**P**

**IITRCS01**

**IITRCS06**

**IITRCS07**

**IITRCS11**


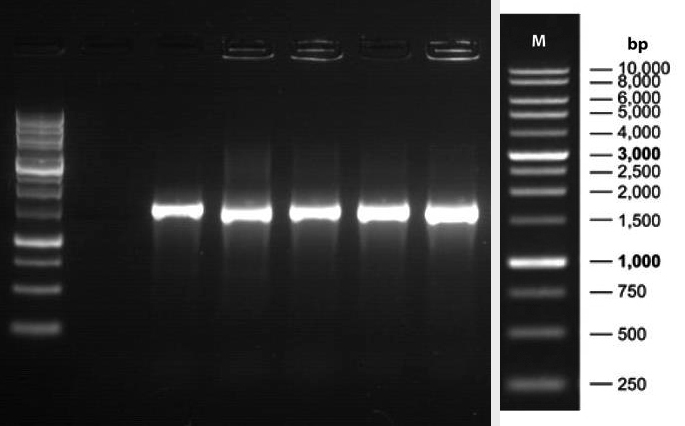


**Supplementary Fig. S1** PCR amplification of 16S rRNA gene of isolated bacterial strains; Lane M: 10,000 bp DNA ladder; N: PCR positive non-template control (water only); P: positive control (DNA extracted from *E. coli* used as template)


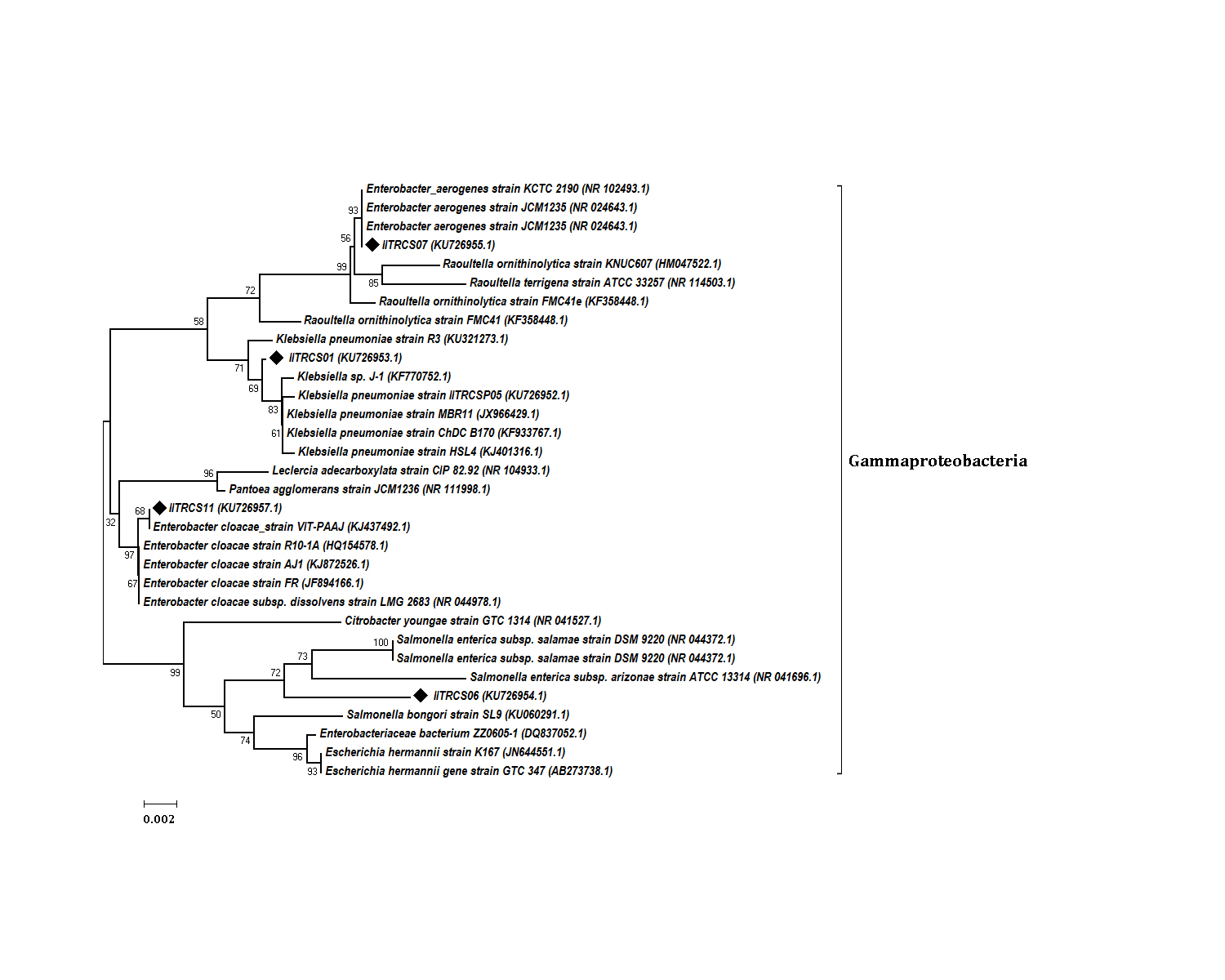


**γ-Proteobacteria**

**Supplementary Fig. S2** Phylogenetic tree showing the inter-relationship of isolated bacterial strains with the most closely related species inferred from sequences of 16S rRNA gene. The tree was generated using Neighbour-Joining method with a bootstrap value of 1,000 replicates. The scale bar shows 0.002% sequence divergence.

**Supplementary Fig. S3** Growth pattern of developed bacterial consortium during degradation and decolourisation of SGA-MRPs


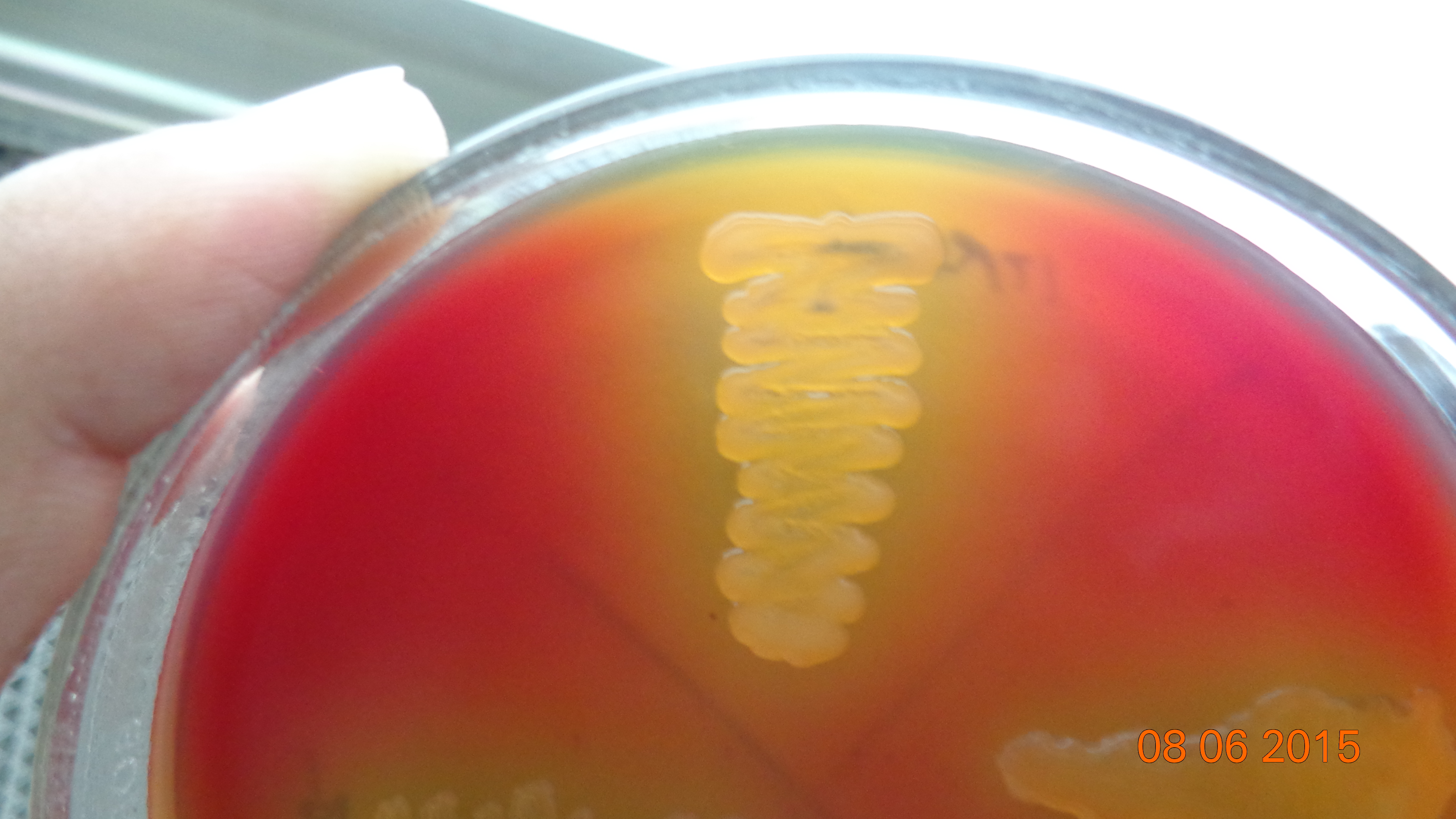


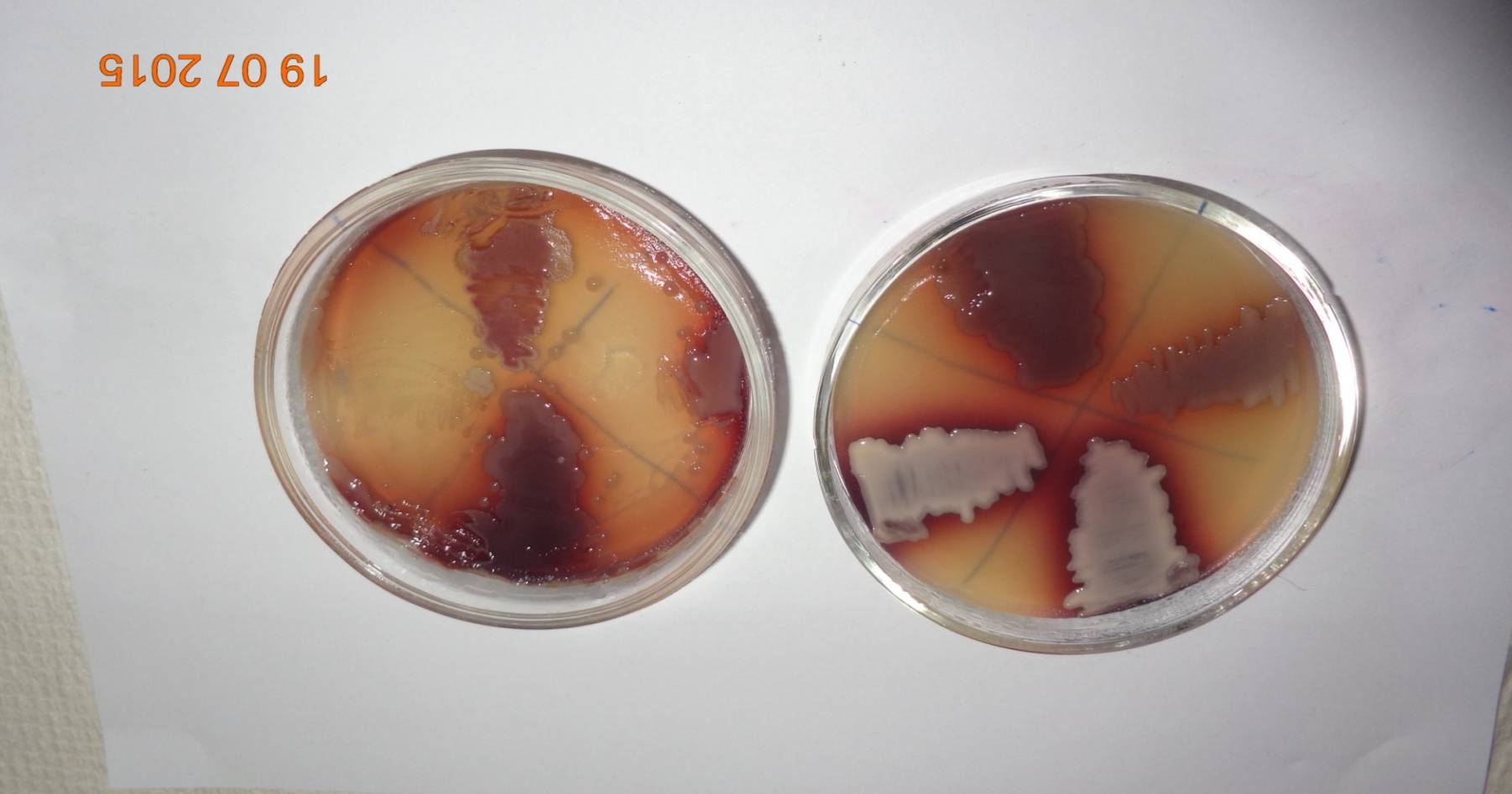


**Supplementary Fig. S4** Manganese peroxidase and laccase activity (a) on phenol red amended GPM agar plate (b) guaiacol amended B&K agar plate (c) in broth assay during decolurisation and degradation of SGA-MRPs


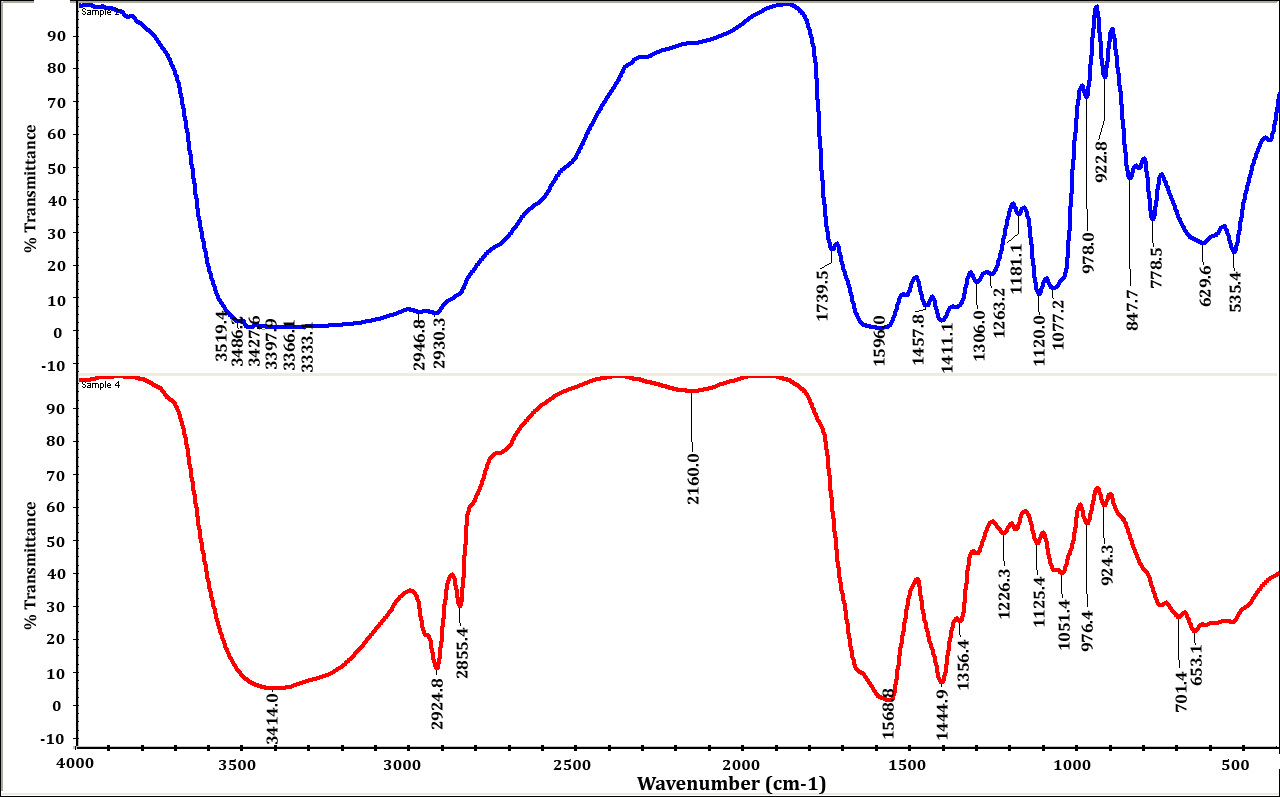


**Supplementary Fig. S5** FT-IR spectra of SGA-MRPs (a) untreated (b) bacterial treated after 192hrs

**Supplementary Fig. S6** Effects of various concentrations of untreated and treated SGA-MRPs on the seed germination and seedling growth of *Phaseolus mungo* L. (a) percent germination (b) germination index (c) phytotoxicity percent (0%: tap water used as a control)
